# Current Perspectives on B Lymphocytes in the Immunobiology of Hepatocellular Carcinoma

**DOI:** 10.3389/fonc.2021.647854

**Published:** 2021-06-21

**Authors:** Miaomiao Qin, Danping Wang, Yijiao Fang, Zhiying Zheng, Xinyang Liu, Fan Wu, Liangliang Wang, Xiao Li, Bingqing Hui, Shijie Ma, Weiwei Tang, Xiongxiong Pan

**Affiliations:** ^1^ Department of Anesthesiology, The First Affiliated Hospital of Nanjing Medical University, Nanjing, China; ^2^ Department of Oncology, The First Affiliated Hospital of Nanjing Medical University, Nanjing, China; ^3^ Department of Anesthesiology, Fudan University Shanghai Cancer Center, Shanghai, China; ^4^ Department of General Surgery, Nanjing First Hospital, Nanjing Medical University, Nanjing, China; ^5^ Department of Gastroenterology, The Affiliated Huaian No.1 People’s Hospital of Nanjing Medical University, Huaian, China; ^6^ Hepatobiliary/Liver Transplantation Center, The First Affiliated Hospital of Nanjing Medical University, Key Laboratory of Living Donor Transplantation, Chinese Academy of Medical Sciences, Nanjing, China

**Keywords:** hepatocellular carcinoma, B lymphocyte, immunity, tumor-infiltrating B lymphocyte, immunotherapy

## Abstract

Immune cells infiltrating tumors are capable of significantly impacting carcinogenesis through cancer promotion and anticancer responses. There are many aspects of hepatocellular carcinoma (HCC) related T lymphocytes that are undergoing extensive studies, whereas the effect exerted by B lymphocytes remains a less researched area. In this study, the latest research on the effect of B lymphocytes as they infiltrate tumors in relation to HCC is presented. Their prognosis-related importance is analyzed, along with their function in the tumor microenvironment (TME), as well as the way that B cell biology can be employed to help create a B cell therapy strategy for HCC.

## Introduction

Hepatocellular carcinoma (HCC) refers to the most common form of major HCC, the sixth most common form of cancer worldwide (1), and the third most significant factor of cancer-associated mortality (2). In nations with highly prevalent hepatitis B virus, hepatitis B virus (HBV) vaccination has been suggested to decrease the incidence of HCC (3). Surgery, locally-related treatment and whole-body therapeutic processes have been regularly employed to treat HCC. The method to determine the optimal treatment for cases is determined by numerous parameters (e.g., the status of the case, the presence of vascular infiltration, the tumor’s extent and size, as well as the severity of the underlying liver disease) (4).

Existing studies have strengthened the idea that cross talk and reciprocal signaling of liver micro-scale environments and malignant cells promote tumorigenesis and HCC pathogenesis. Such a dynamic network in which tumor cells grow is termed as the tumor microenvironment (TME). In HCC, TME is immunosuppressive, thereby inducing tolerance and promotes the proliferation, invasion, and metastasis of tumors. The mentioned tumorigenic response is mediated from environmental cues, carcinogenic processes, several immunosuppressive cell subsets, inflammation-related molecules and signaling channels (5–7). Immunotherapy is gradually emerging in tumor treatment, especially immune checkpoint inhibitors (ICIs), which have suggested an encouraging efficacy that has changed the treatment model of advanced HCC. Subsequent research on the immunobiology of HCC may contribute to the development of targeted therapies to reduce mortality (8). At this stage, the research on the biological characteristics of T cells is increasing in an exponentially increasing. As opposed to the mentioned points, the insights into B cell biology related to cancer are not complete. B cells that can infiltrate tumors are able to be identified at various phases of HCC development, and their existence varies in accordance with the stage and histology-related sub-category. Since they impact humoral and cellular immunization, manipulation of B cell biology can provide significant immunotherapy opportunities (9, 10). Therefore, it is essential to gain insights into the B cell biology of HCC for resetting the immunity environment of the cancer micro-scale environment for the treatment of HCC. The present study concludes the latest studies of B cells and stresses the dual biology-related influences exerted by B cells. Furthermore, the subsequent therapy-related strategic procedures related to HCC therapy are studied in depth.

## B Cells Development

Hematopoietic stem cells in the bone marrow account for continuously producing B cells. B cells are rigorously controlled through hematopoietic precursors based on the gene regulating network, remodeling of chromatin, cytokine signal transduction, and the affinity selection *via* B cell receptors (BCR) (11). B cells are guided by the chemokine CXCL13 as they migrate into secondary (or peripheral) lymphoid tissues (e.g., the spleen, lymph nodes (LNS) and follicles in Peyer’s plaques (PPs)) (12). The germinal center (GC) in the secondary lymphoid organs (SLOS) initiates B cell activation for the signal sent by BCR when it encounters antigens. Besides BCR, the most complex antigens bind to other receptors on B cells (e.g., toll-like receptors (TLRs) or complement receptors (CR2s)) (13). After being activated, B cells undergo Isotype Switching, thereby altering the isotype of BCR from IgM and IgD to IgG, IgA, or IgE. Subsequently, the converted B cells then produce long-lived plasma cells or memory B cells to participate in the anti-tumor response (14).

Inconsistent with conventional B cells (B2 cells), B1 cells are one uncommon subgroup of B lymphocytes which can spontaneously secret immunoglobulin M (IgM) and critically impact innate immunity (15). It is evidenced that B1 cells expedite the metastasis of malignancy-related melanomas and induce the development of chronic lymphocytic leukemia (16, 17).

Tertiary lymphoid structures (TLSs) are lymphoid aggregates formed in non-hematopoietic organs that respond to non-chronic hematopoiesis (e.g., infections, transplant rejections, autoimmune diseases and cancers) (18). TLS can act as an SLOS to exert anti-tumor effects by producing plasma cells (19). TLS are induced in early hepatic lesions (EHL) (20), and TLSs are related to the risk of early postoperative HCC recurrence, as reported in several reports (21, 22).

### Function of B Cells

On the whole, B cells are involved in antigen presentation and the secretion of specific antibodies. The T cell immune response is activated by B cells. Antigen-specific interactions require antigen internalization *via* BCR and should then be delivered to T cells by employing an MHC-limited method (23). B cells act as plasma cells with long lifetimes, which can generate antigen-specific antibodies (24). CD20–CD79α + B cells are producers of antibodies, which can secrete IgA, IgG and IgM. Moreover, the mentioned cells can act as APCs and express some costimulatory molecules (e.g., CD80 and CD86). This has been reported to display a close relationship to a better prognosis of liver cancer (25).

### Regulate Immune Responses by Producing Cytokines

The function of B cells has long been explained in an antibody dependent manner (26). There is now increasing evidence that B cells have a noticeable effect on the regulation of innate immunity and adaptive immunity by producing cytokines (27, 28).

**Figure 1:**
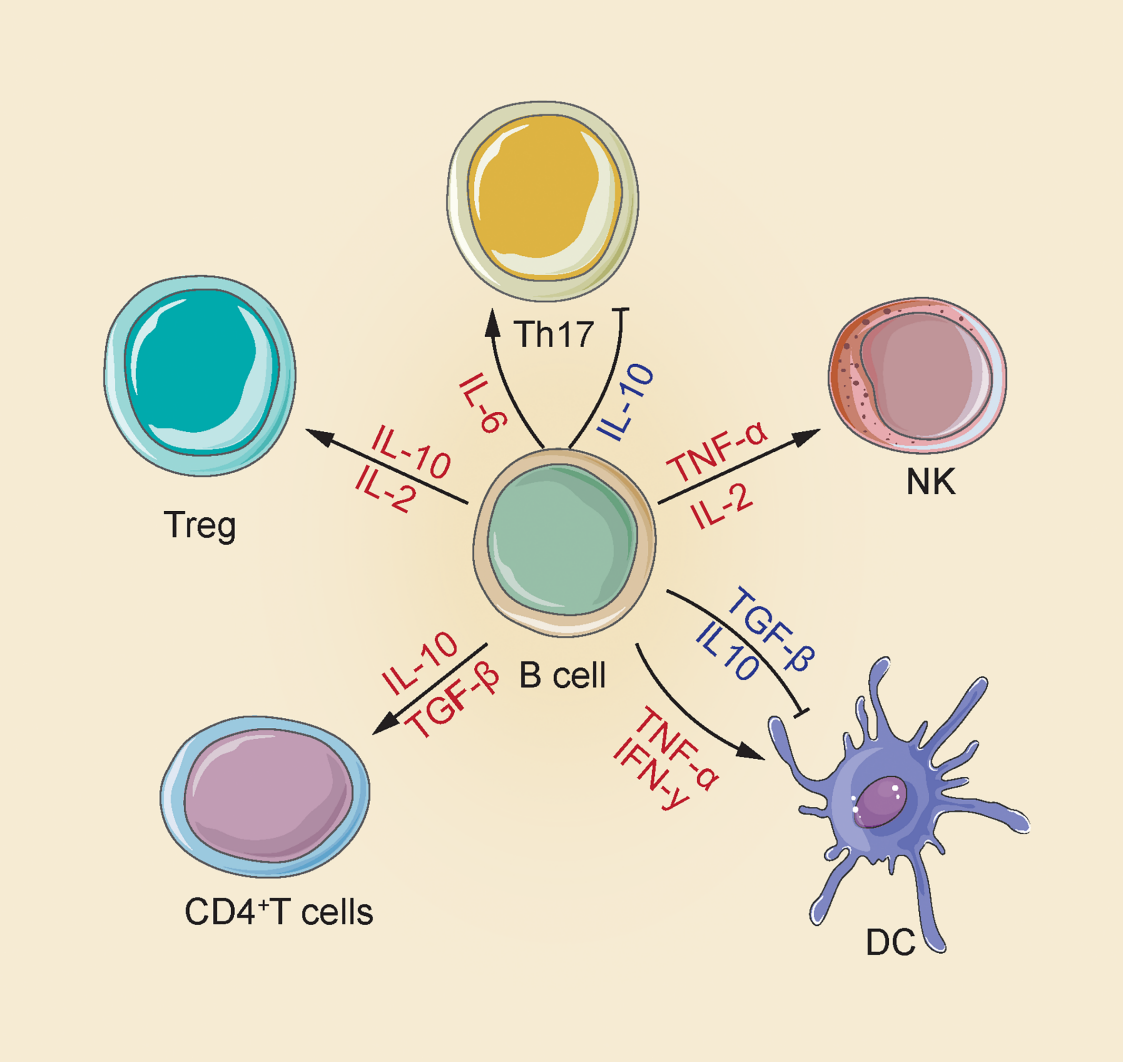
There are multiple mechanisms for the positive and negative effects of B cells produced by cytokines in the immune response. B cells increase NK cell activation and Treg differentiation by releasing IL-2 and TNF-α; releasing of IL-2 and IL-10 to enhance Treg differentiation; releasing of IL-10 and TGF-β CD4^+^ T cell apoptosis and inhibit DC maturation, and also secrete IFN-γ and TNF-α to induce DC maturation; release IL-10 to inhibit Th17, release IL-6 to enhance Th17 response (33).

B cells are capable of synthesizing several cytokines that exert a disease-causing/protecting effect on malignant tumors, infection and autoimmunity (29, 30). The most representative immunosuppressive cytokines include transforming growth factor (TGF)-β and Interleukin (IL)-10 that can negatively regulate the immune response by suppressing Th cell responses, limit the Th1 cell and Foxp3+ Treg differentiation, reduce APC functions and pro-inflammation-related cytokine release from monocytes, as well as cause CD4+ T cell death and CD8+ T cell anergy (31, 32). In addition, cytokines playing a positive immunoregulation role consist of pro-inflammatory cytokines (e.g., IFN-α, IFN-γ, TNF-α and IL-1 IL-2, IL-6, IL-8, IL-12, IL-16 and IL-35); Th2 cytokines (IL-13, IL-5 and IL-4); macrophage colony stimulating factor (M-CSF); granulocyte macrophage colony activating factor (GM-CSF) and hematopoietic growing factor granulocyte colony stimulating factor (GCSF); even chemokines (e.g., CCL7 and CCL5). Specific mechanisms are presented below: IL-2, 1L-4, IL-12 and IFN-αcapable of promoting Th1/2 and Th17 development and responses; GM-CSF triggering neutrophil response; IFN-α, TNF-α and lymphotoxin(LT)-α/βtriggering DC maturation and forming processes of lymphoid configurations; IFN-α improving the NK cells and macrophage activation, stimulating their own development, and promoting antibodies production (**Figure 1**) (33).

In malignant tumors, self-reactive and pathogen-infected B cells are capable of spontaneously producing cytokines, thereby exerting pathogenic or protective functions (34). According to Song et al., Bregs can cause dysfunction of DCs and increase of Foxp3(+) Treg, which critically impacts the progression of liver cancer (35).

### Double Aspects of the Biology-Related Function Exhibited by B Cells

The development of tumors involves a complex biological procedure, which covers the dynamic relationship of tumors and immune system cells (36). T cells have always been considered the main participants in the anti-tumor immune response. T cells cannot work alone, and the optimal adaptive immune response requires B and T lymphocytes to be activated in a coordinated manner (37, 38). However, B cells are commonly ignored in tumor immunological studies, despite their very complex role in the host’s immune response toward malignant tumors.

### Promoting Tumor Progression by B Cells

A study performed nearly 60 years ago reported that a lack of B cells impairs the tumors’ development, which initially suggested that B cells are likely to enhance the growth of tumors (39). Since then, significant number studies have revealed the contribution made by B cells to facilitating tumor development. According to independent studies on multiple mouse cancer models, B cells are related to accelerated tumor growth. Some of the mentioned reported that B cells boost the development of squamous cell cancer by activating Fcγ receptors (FcγR) on myeloid cells, and the cells expedite the development of melanoma and lung cancer by facilitating the formation of new blood vessels in tumors (40, 41). Complement C5a refers to a vital part of humoral immunity, which impacts the development of cancer. It has previously been reported (42) that complement C5a promotes the occurrence of squamous cancer and limits the response of T cells to chemotherapy. Ammirante et al. (43) found that lymphotoxin originating from B cells activates prostate cancer resisting castration. In human tumors, antibodies lead to the production of circulating immune complex (CIC) (44), thereby displaying an association with the weak prognostic process of cases with pancreatic ductal adenocarcinoma. Given the reports, TIL-B cells promote the bladder cancer metastasizing process by modulating the signals of IL-8/androgen receptor (AR)/MMPs (45).

Besides the above non-particular tumorigenic activity, the tumor-enhancing capacity of B cells stems from the primary mediating process *via* various B cell populations called regulating B cells or Bregs. A major characteristic of Bregs is that they inhibit the cytokine production (e.g., TGF-β, IL-35, and IL-10) (46). IL-10 can inhibit other stimulating cytokines’ production, causing a decrease in the reactivity of NK cells, Th1 cells, and CD8 + T cells. The TGF-β production can drive the conversion of CD4 + T cells into Tregs, thereby inhibiting Natural Killer (NK) cells and CD8 + T cells, which are critical to inhibit the growth of tumors (47–49). Lindner et al. (50) reported that interleukin 21 facilitates the infiltration of granzyme B-expressing B cells into tumors, while inhibiting the Foxp3 expression in Tregs and CD4 + T cell proliferation by secreting TGF-β and IL-10. The mentioned event is common in breast cancer, ovarian cancer, colorectal cancer, cervical cancer, and prostate cancer. In a study of cases involving acute myeloid leukemia (AML), the authors reported (51) that Breg cells were more highly concentrated in AML cases within the bone marrow (BM) and peripheral blood (PB) in comparison to those in HDs. For AML cases, the amount of Breg cells was elevated, which likely indicates weak prognostic results. It was demonstrated that the tBregs were capable of transforming nonregulating T cells (non-Tregs) into active Tregs based on the secretion of TGF-β, thereby suppressing the proliferation of T cells and increasing the tumor metastasis. In addition, cancer cells could convert normal B cells into tBregs. Thus, if cancer persists, the cancer cells were likely to cause tBregs to generate and suppress the antitumor immune response (52, 53).

### Anti-Tumor Functions and Prognostic Value of B Cells

There is a lot of evidence which suggests that B cells can interact with tumor cells in a direct manner, or indirectly enhancing anti-tumor immunity by assisting other immune functions. In the case of cancer, B cells stimulated from tumor cells will generate antibodies involved in anti-tumor immunity, causing a strong humoral response. According to a study (54), the allogeneic tumor rejection is performed in mice through the natural appearance of IgG antibodies that bind to tumors, thereby enabling dendritic cells (DCs) to internalize tumor antigens and then causing T cells to react to tumors. They used the mentioned system to successfully eradicate tumors in rat melanoma, pancreatic cancer and lung cancer models. Tumor-draining lymph nodes (TDLNs) B cells conferred strong humoral responses to tumors. By producing IgM, IgG and IgG2b, tumor cells are bound specifically, and tumor cell lysis is caused in the presence of complement (55). They are also capable of conducting anti-tumor processes by recognizing tumor-particular antigens and facilitating the production of antibodies, the antigen presenting cell (APC) function, or directly killing carcinogenic cells (56). In the long-term immune response between tumors and the immune system, the number of dendritic cells is small, and B cells act as local APCs on a micro scale within tumors, thereby facilitating the survival and proliferation of T cells. For instance, in ovarian cancer, CD20 + B cells can be found in the vicinity of T cells, which indicates that they play a role in the effect exerted by APC in this case (57). B cells can directly kill tumor cells, and after stimulation with CpG-containing oligodeoxynucleotides (CpG ODN), CD19 + PBMC (B cells) can kill tumor cells (58).

In many human malignancies, the B cell infiltration process shows a relationship to a more positive prognostic result (59). It has previously been reported (60) that CD20+ lymphocytes (a B lymphocyte antigen expressed on mature B cells but not expressed on plasma cells) are effective at promoting good outcomes in a wide range of human cancers, including breast cancer, cutaneous melanoma, and HCC (61). The concentration of tumor infiltrating B lymphocytes (TIBs) is an independent prognostic factor in metastatic renal cell cancer (mRCC) patients, as well as a prediction-related marker for the tyrosine kinase inhibitor (TKI) therapy response (62). The role of B cells in the development of HCC is consistent with the mentioned types of malignant tumors, but there are also special features.

## B Cells and Hepatocellular Carcinoma

### Hepatitis Virus and B Cells

As the main cell-related part in the TME, the distributing process, frequency, and prognosis-related importance exhibited by invasive B cell subsets within HCC is still controversial. Virus-related hepatitis and the occurrence of HCC show an inseparable relationship (63). An early report stated that the hepatitis C virus (HCV) can infect liver cells by binding to B cells in peripheral blood, increasing the risk of HCC (64). Besides, some research indicated that the interaction between HBV and B lymphocytes can be explained by the humoral immune deficiency in individuals who develop HCC (65). According to another study, the loss of memory B lymphocytes in chronic HBV infection may play a role in the persistence of HBV infection and the development of HCC (66). As revealed from the mentioned studies, the relationship between B cells and liver cancer are inconsistent with other tumors.

### Tumor-Infiltrating B Lymphocytes in HCC

**Table 1** (66–77) summarizes the studies that have identified TIL-B in tissues and examined its prognostic significance. These studies primarily concentrated on HCC, and immunofluorescence histochemistry and flow cytometry were mostly used to identify the number and distribution of lymphocytes in tumor tissues. As reported by Zhao et al. (67), compared with non-tumor liver tissues, all B cell subpopulations in tumors were reduced. To be specific, native B cells (Bn) and CD27-isotype-switched memory B cells (CD27-SW Bm) acted as the independent prognostic factors for HCC survival, and high concentrations of tumor infiltrating B cells led to more effective clinical results. However, as indicated by Faggioli et al., the presence of infiltrating B cells is related to increased tumor invasiveness and decreased disease-free survival in human HCC (68). As indicated from **Table 1**, multiple subgroups of B cells coexist in the tumor microenvironment of HCC and exert dual effects on tumors. CD20+ B cells are the most abundant in TLB, and mature CD40+ plasma cells, natural B cells, and CD27+ memory cells can also exert anti-tumor effects. Bregs, producing IL-10, primarily promote cancer. There is currently no clear surface marker for Bregs. B cells mainly exist in the marginal areas of tumors, in which there are relatively few infiltrating B cells (75).

**Table 1:** Tumor infltrating B lymphocytes in human hepatocellular carcinoma.

Study	Cell subsets	Tumor Classification	Methods	No. of cases	Outcome and prognostic significance
Zhang, Ma (67)	CD27 ^-^ Sw Bm Bn	HCC	IHC	619	high density of tumor-infiltrating B cells are significantly and independently associated with better survival rates.
Faggioli, Palagano (68)	CD20^+^	HCC	IHC	116	presence of infiltrating B cells correlated with increased tumor aggressiveness and reduced disease-free survival in human HCC.CD20^+^B cells that produce TNF-α limit senescence, which is beneficial to the progress of HCC.
Garnelo Tan (69)	CD20^+^ CD27^+^ CD38^+^ CD40^+^	HCC	IHC PCR FACS	112	the close proximity of tumor-infiltrating T cells and B cells indicates a functional interaction between them that is linked to an enhanced local immune activation, contributing to better prognosis for patients with HCC.
Brunner, Itzel (70)	CD20^+^ CD79a^+^	HCC	Genetic analysis	2,158	high levels of immunoglobulin fragments identified in gene expression analysis were detected in patients with high-density B cell infiltration.
Schneider, Teufel (71)	Igh6	(DEN) induced liver cancer mice HCC	IHC FACS Genetic analysis	15 139	adaptive immune cells can strictly control the occurrence of liver cancer, and B cells seem to be mainly involved in restricting tumor growth.
Shi, Gao (72)	CD20^+^	HCC	IHC FACS	120	high density of marginal infiltrating B lymphocytes (MIL-B) was positively correlated with smaller tumor size, no vascular infiltration, and increased CD8^+^ T cell density. Determined as an independent prognostic factor for patients with hepatocellular carcinoma.
Ding, Xu (73)	CD20^+^	HCC	Meta-analyses	452	patients with high-density CD20 ^+^ B cells at the edge of the tumor have higher disease-free survival and overall survival.
Shen, Xu (74)	CD40^-^	HepG2	cell culture *in vitro*	/	CD40^-^B cells can activate CD8 ^+^ T cells that produce antigen-specific interferon gamma, exerting a killing effect on HepG2 cells.
Wang, Wang (66)	CD27^+^	HCC	FACS	38	The percentage of memory B lymphocytes decreases with the progression of HCC.
Xiao, Lao (75)	PD-1^hi^B cell	HCC	IHC FACS ELISA	53	PD-1 hi B cells act through IL10-dependent pathways after interacting with PD-L1, thereby causing T cell dysfunction and promoting disease progression.
Liu, Wei (76)	CXCR3^+^B cell	HCC	IHC FACS ELISA	40	The selective recruitment of CXCR3(+) B cells bridges the pro-inflammatory interleukin 17 response and the polarization of tumorigenic macrophages in the tumor environment, blocking the migration or function of CXCR3(+) B cells may help defeat HCC.
Wang, Wang (77)	CD19^+^ CD5 ^+^ CD1d ^hi^ B regs	PHC	IHC	/	Tregs and Bregs inhibit anti-tumor response, and all these cells may promote the development and progress of PHC together.

### Anti-Tumor Functions: HCC

As mentioned above, B cells exert an anti-tumor effect in a wide range of tumors. A question is raised that how do B cells exert such an effect in HCC occurrence and development. HCC refers to a frequently occurring cancer with a weak prognostic outcome and a low 4-year survival ratio. The powerful and valid cytotoxicity under the mediation of CD4 + T cells is related to a higher HCC survival rate as well as a lower HCC reoccurrence ratio (78). According to a study conducted by Zhang et al. (67), Bn and CD27-Sw Bm show a significant positive correlation with a higher survival rate. In fact, BN is capable of expressing CD80, CD86, CXCR3, CCR5 and PD1, thereby helping activate T cells. In addition, CD27-Sw Bm can produce IgG or IgA to promote humoral immunity. Garnelo et al. (69) reported that the interaction between B cells and T cells leads to their activation, which is also essential for the control of HCC. The density of TIB is related to the number and activation state of T cells and NK cells, and is consistent with reduced tumor cell viability. In a mouse model transplanted with Hepa1–6 cells, B cell depletion results in an enhanced tumor growth and a decreased local T cell activation. To be specific, the expressions of IFN-γ, granzyme B and CD69 on CD4 + T cells are significantly down-regulated, and on CD8 + T cells, PD-1 increase significantly. Besides, CD27 and CD40 costimulatory molecules and TILs expressing the stimulation marking element CD38 in tumors are related to patient survival. Shen et al. (74) used CD40 ligand-activated B (CD40-B) cells in an electroporation of overall RNA in HepG2 cells as an alternative to APC to induce a particular CD8 + T cell responses. They found that the total RNA derived from CD40- B cells under the electroporation in cancer cells can also act as an alternative to APC for inducing antigen-particular CD8 + T cell responses, and these cells can therefore be employed for HCC immunotherapy. According to the unsupervised gene expression analysis of the complete cancer transcriptome by Brunner et al. (70) (N = 2158), the infiltration of CD20 cells and CD79a cells at the edge of the infiltration caused the prolonged survival of patients. In addition, high levels of immunoglobulin fragments reported in gene expression analysis were detected in patients with high-density B cell infiltration. The mentioned study initially proved that B cells may directly produce anti-tumor effects by secreting immunoglobulins, and are therefore beneficial to HCC patients. Moreover, another study observed tumor-related B cell activation and the production of antigen-specific immunoglobulins in the occurrence of DEN-induced liver cancer. It was stipulated that when tumors are formed and inflammation is triggered by the tumor itself, B cells can inhibit tumor growth (71). In addition, CD20 ^+^ B cells were suggested to be concentrated around tumor deposits and to form a dense cell layer in the marginal area of the infiltration. According to Shi et al., TIL-B stimulates CD8 ^+^ T cells by producing high levels of IFN-γ and IL-12p40, and exerts a direct killing effect (72). B cells act as antigen presenting cells (APCs), and they are capable of stimulating T cell responses. Besides T cells, according to Jeannet et al. (79), the lack of B cells can reduce the survival rate of NK cells in the spleen, as well as the direct killing function against tumor cells.

**Figure 2:**
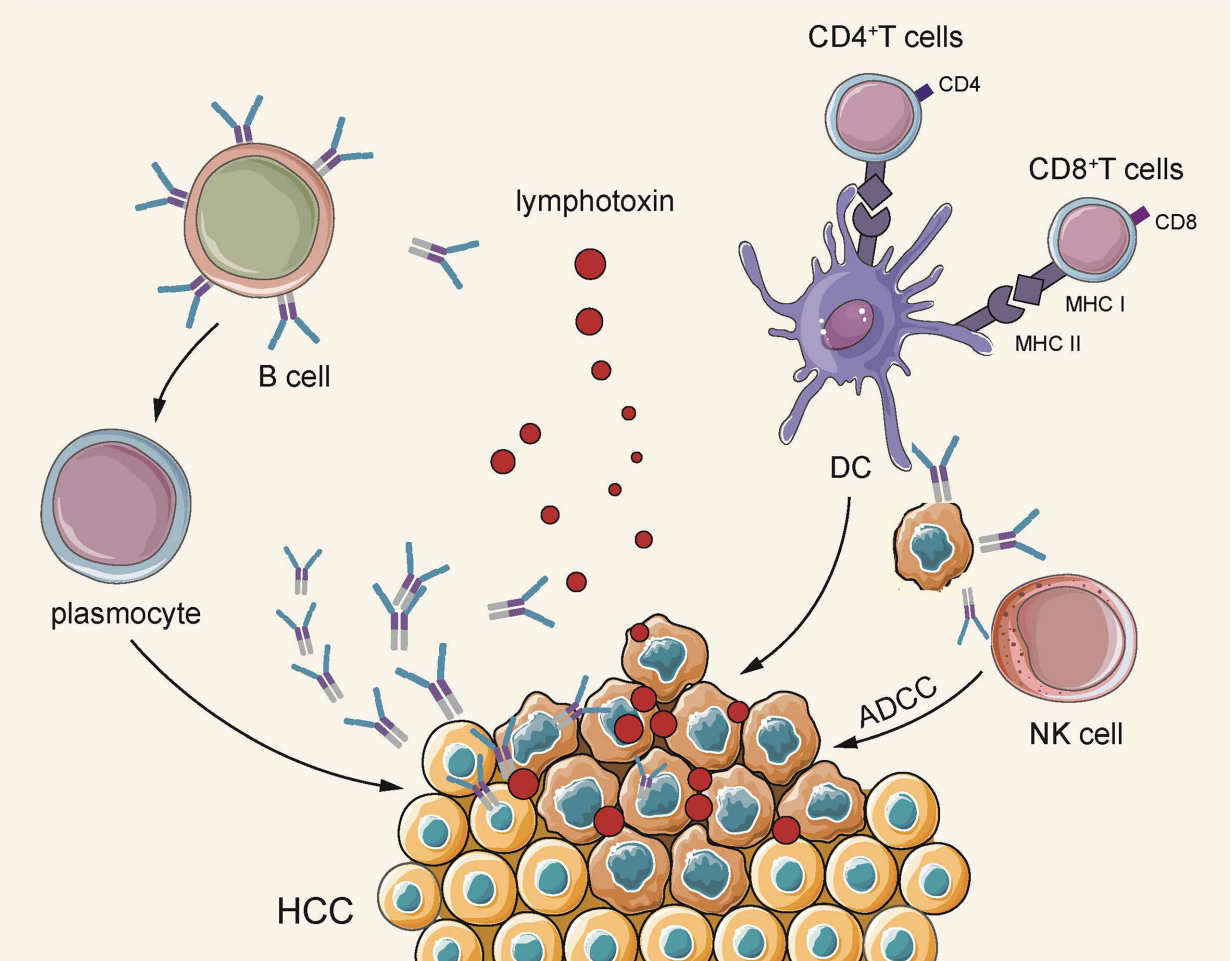
On the one hand, B cells secrete lymphotoxin through TLS, and plasma cells secrete antigen-specific antibodies to directly kill tumors. On the other hand, they indirectly play an anti-tumor effect by acting as APC to activate cellular immunity and activate the natural killing effect of NK cells.

In brief, B cells are distributed simultaneously in multiple subgroups of HCC, and different subgroups exhibit an anti-cancer effect at different stages. On the whole, B cells secrete specific antibodies to directly act upon tumor cells to exert humoral immunity, acting as APC to promote T cells to participate in cellular immunity, and also stimulate NK cells to directly kill tumor cells. Furthermore, B cells are responsible for secreting pro-inflammatory factors, thereby stimulating T cell activation (**Figure 2**).

### Pro-Tumor Functions: HCC

As mentioned above, B cells can play a tumor-promoting role in squamous cell cancer through complement and lymphotoxin (42, 43).In the tumor microenvironment of HCC, how do B cells exert the cancer-promoting effects?

### Bregs in HCC

**Figure 3:**
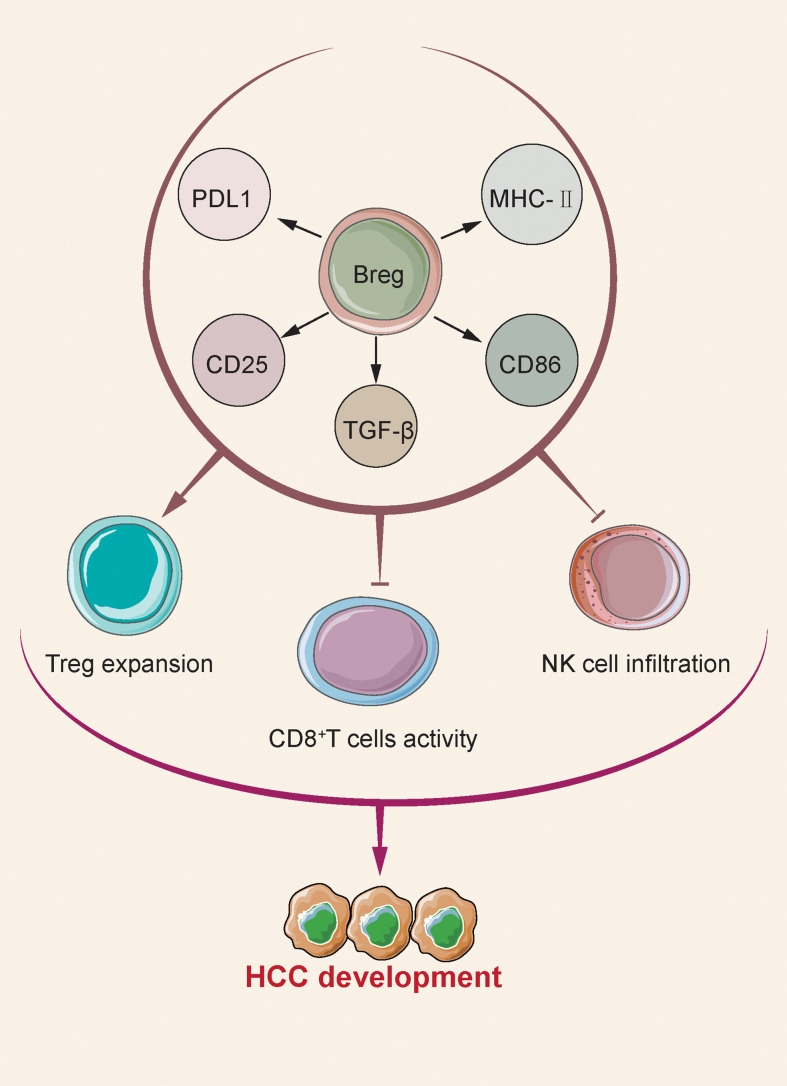
Bregs regulates other immune cells, affecting tumor development. After B cells migrate to the tumor bed, they develop a Breg phenotype and increase the expression of membrane-bound TGF-β, CD25, MHC-II, and costimulatory ligands CD86 and PD-L1. Breg supports Treg expansion, inhibits IFN-γ production and CD8 + cytolytic T cell activity, thereby inhibiting anti-tumor response and leading to enhanced tumor growth.

It is worth mentioning that B cells can display an association with pro-tumorigenic processed by stimulating myeloid-derived suppression cells (MDSCs), producing pro-tumorigenic cytokines, and stimulating immunosuppressive regulating T cells (56). The pro-tumoral process undergoes major mediation by regulating B cells (Bregs). B cells may drive tumor progression by up-regulating a range of gene expressions that drive tumorigenesis, or by reducing the immune response. Bregs, a novel descriptor subset of B cells, has been shown to exert an inhibitory effect on the immune system (**Figure 3**). Bregs can suppress other immune cells based on cytokine secretion and the antigen presenting processes, so they can impact the origin and development of autoimmune diseases and cancer. The existing proposed standards are unclear for identifying Bregs (80). Specific to breast cancer, ovarian cancer, colorectal cancer, cervical cancer and prostate cancer, Bregs exert immunosuppressive function by secreting IL-10 and TGF-β to promote tumor development (50). B cells expressing IL-10-can inhibit cytotoxic CD8 ^+^ T cells, inflammation of T helper 1 (Th1) cells and Th17 cells, thereby facilitating the differentiation of T (Treg) cells (81) regulation.

The phenotype, function and clinic-related relevance of Bregs cells in human HCC have been rarely investigated. Chen et al. (82) found that frequencies of peripheral Tregs and Bregs in HCC cases were elevated after surgery and showed that the circulating Bregs of CD19 ^+^ CD24 hi CD38 hi directly promote the progression of HCC through the CD40/CD40L signaling pathway. Lei et al. (83) found that after studying sorafenib in HCC treatment, the prognosis of patients with advanced HCC with a reduced Bregs ratio was better than that of patients with a constant or increased Bregs ratio. The proportion of Bregs in peripheral blood can be considered an early biological indicator for predicting the efficacy of sorafenib. According to Shao et al. (84), a noticeably higher percentage of B cells were reported at the edge of tumors than within the tumor and non-tumor areas. The percentage of circulating Bregs in HCC cases was significantly higher than that in the healthy controls. The increase in circulating blood Bregs is related to tumors in their advanced stages, a greater diversity of tumors, and venous infiltration, which has been verified in animal experiments. Lastly, it was found that Bregs directly interact with HCC cells through CD40/CD154 signaling, thereby promoting the growth and invasion of HCC.

Besides directly acting on tumor cells, the critical way for bregs to promote cancer is to inhibit T cell function. It has been indicated that PD-1 hi B cells are the main subtype of Bregs in human HCC and operate through an IL10-dependent pathway to induce T cell dysfunction, thereby creating conditions conducive to tumor progression (75). Moreover, according to Xue et al. (85), compared with healthy controls, the Bregs’ frequency in HCC cases was noticeably higher. Bregs and CD4^+^ cytotoxic T cells were observed to be negatively related in cells infiltrating tumors. Ye et al. (86) used flow cytometry to analyze the level of TIM-1Breg cells in samples from 51 cases of HCC. In addition, as indicated from the phenotype and function, compared with matched tumor tissues, the infiltration of TIM-1Breg cells in tumor tissues in cases of HCC was significantly higher. The infiltrated TIM-1Breg cells showed the CD5CD24CD27CD38 phenotype, high levels of immunosuppressive cytokine IL-10, as well as the strong inhibitory activity toward CD8^+^ T cells. The accumulation of TIM-1Breg cells’ in tumors is related to the late stage of the disease, the assessed HCC’s early recurrence as well as the down-regulated survival rate of HCC cases.

Bregs and Tregs are closely related to each other. Wang et al. (77) investigated the interaction between Th17 and tumor cells in the TME of major HCC, and the relationship of Th17, the regulation for T cells, and the regulation for B cells. The increase in blood Th17 corresponds to the progression of tumor lymph node metastasis (TNM) stage, and Th17 increases simultaneously with T and B in HCC. All of these weaken anti-tumor responses and act synergistically with tumor growth, thereby accelerating immune escape in the TME.

### Other Types of B Cells in HCC

Faggioli et al. reported that CD20^+^ B cells could affect the polarization of macrophages through Fcγ receptor-dependent signal transduction, directly stimulating fibroblasts, or inhibiting metalloproteinase activity through antibody secretion. The high infiltration of CD20^+^ B cells is related to the adverse effects of liver cancer (68). Ouyang et al. (87) reported that more than 50% of B cells in HCC exhibit a low/activated phenotype of FcγRII, and the high infiltration of these cells is positively correlated with cancer progression. In the TME, the early activation of monocytes is important for semi-mature dendritic cells and FcγRII low/activated B cell production. It was previously reported that CD40L B cells transfected with total tumor RNA isolated from HCC cells can induce cytotoxic T cell responses *in vitro* (88).

As indicated from many of the mentioned studies, the tumor-promoting effect of B cells is mainly attributed to the regulation of B cells that secrete IL-10 as their immunosuppressive function. Bregs induce the apoptosis of T cells, hinder the maturation of DCs, inhibit cellular immunity, as well as expedite the occurrence of HCC.

## Conclusion and Perspectives

As impacted by the complex distribution of B cells observed in existing studies on peripheral blood and tumor microenvironment, the cells are suggested to play a contradictory role in tumor development. On the one hand, B cells play a humoral immune role through well-known antibodies. They act as an APC and help T cells since they enhance the function of cellular immunity and also directly kill tumor cells. Their anti-tumor ability has been confirmed extensively. In addition, B cells play a noticeably complex role *via* their seemingly insignificant innate immunity, which is relatively weak. Bregs that produce IL-10 have a very strong immunosuppressive function, and B cells can also secrete cytokines to act on other immune cells and have a significant immunomodulatory function on DC cells, NK cells, neutrophils, and so on. Thus, B cells are allowed to directly promote tumor development under certain conditions and indirectly interfere with tumor immunotherapy through other infectious and autoimmune diseases. As impacted by the complex role of B cells mentioned earlier in this article, it is important to distinguish the multiple functions of B cell subpopulations, as the mentioned cells are likely to be targets for tumor immunotherapy. The concept of Bregs is still unclear, since no phenotypes or transcribing elements exist (e.g., Tregs’ FOXP3). The way B cells act as regulators of Bregs remains unclear. If the inflammation is a major condition for achieving differentiation within Bregs, the complex and powerful immunomodulatory functions of Bregs and the feasibility of isolating the stimuli necessary for inducing B-cell differentiation are still unclear. Furthermore, mouse models employed to investigate cancer are commonly accompanied with a quick response (e.g., viral infections), which are not likely to accurately represent the chronic anti-carcinoma immune responses observed within humans. For the mentioned reason, major breakthroughs are still required to discover the mystery behind B-cell-based immunotherapy.

HCC occurrence and development are related to chronic inflammatory micro-scale environments and immune cell infiltration (10). Both innate immune cells and T cells can directly mediate the development of HCC (89). This study considers that the anti-cancer effect of B cells on HCC is universal, relying on conventional humoral immunity and cellular immunity, and the immune regulation effect of Bregs cannot be ignored. At different stages of HCC development, different subgroups of B cells play different roles, which are determined by the changes in tumor microenvironments. Thus, to refine the distinction of B cell subgroups in subsequent research, the role of other immune cells should be considered; for instance T cells, which coexist with B cells to act upon tumor cells. Bregs research has great potential, and it is believed that a breakthrough in Bregs research in the future will bring new opportunities for the immunotherapy of HCC. Furthermore, the complex relationship among HCC, B cells, and HBV remains unclear, and there may also be novel breakthroughs in the relevant research subsequently.

Lastly, in hepatocellular carcinoma and other cancers, high-scale, prospect-related, and rigorous analysis of B-cell subtype specificity and micro-scale environmental fragment specificity is required to elucidate the effect of B cells on adjusting responsiveness to checkpoint blockades. The mentioned studies will determine the effect exerted by strategies targeting B cells in improving the activity and reducing drug resistance.

## Author Contributions

XP, WT, SM, and BH contributed to the conception of the study. MQ, YF and DW contributed equally to this work. YF and DW organized the table and MQ wrote the manuscript. FW, LW, XLi, ZZ and XLiu contributed significantly to drawing of pictures and manuscript preparation. All authors contributed to the article and approved the submitted version.

## Funding

This work was supported by grants from The Young Medical Talent Funding Project of Jiangsu Provincial Health and Family Planning Commission (No. QNRC2016586).

## Conflict of Interest

The authors declare that the research was conducted in the absence of any commercial or financial relationships that could be construed as a potential conflict of interest.
